# Building communities of practice through case-based e-learning to prevent and manage TB among people living with HIV–India

**DOI:** 10.1186/s12879-022-07957-4

**Published:** 2022-12-29

**Authors:** Reshu Agarwal, Upasna Agarwal, Chinmoyee Das, Ramesh Allam Reddy, Rashmi Pant, Christine Ho, B. Ravi Kumar, Vandana Dabla, Patrick K. Moonan, Melissa Nyendak, Sunil Anand, Anoop Kumar Puri, Sanjay K. Mattoo, Kuldeep Singh Sachdeva, Vijay V. Yeldandi, Rohit Sarin

**Affiliations:** 1 Division of Global HIV and Tuberculosis, U.S. Centers for Disease Control and Prevention (CDC), New Delhi, India; 2 National Institute of Tuberculosis and Respiratory Diseases, New Delhi, India; 3 National AIDS Control Organization, MoHFW, New Delhi, India; 4 Central TB Division, MoHFW, New Delhi, India; 5 Society for Health Allied Research and Education India, Hyderabad, India; 6 ECHO India, New Delhi, India; 7 U.S. Centers for Disease Control and Prevention (CDC), Atlanta, India

**Keywords:** Case-based e-learning, HIV-TB, ECHO, Capacity building, Communities of practice, Integrated care

## Abstract

**Background:**

Co-management of HIV-TB coinfection remains a challenge globally. Addressing TB among people living with HIV (PLHIV) is a key priority for the Government of India (GoI). In 2016, GoI implemented single-window services to prevent and manage TB in PLHIV. To strengthen HIV-TB service delivery, case-based e-learning was introduced to health care providers at Antiretroviral Therapy centres (ARTc).

**Methods:**

We implemented a hub and spoke model to deliver biweekly, virtual, case-based e-learning at select ARTc (n = 115), from four states of India–Delhi, Uttar Pradesh, Andhra Pradesh and Tamil Nadu. We evaluated feasibility and acceptability of case-based e-learning and its impact on professional satisfaction, self-efficacy, knowledge retention using baseline and completion surveys, session feedback, pre-and post-session assessments. We reviewed routine programmatic data and patient outcomes to assess practices among participating ARTc.

**Results:**

Between May 2018 and September 2020, 59 sessions were conducted with mean participation of 55 spokes and 152 participants. For 95% and 88% of sessions ≥ 80% of respondents agreed that topics were clear and relevant to practice, and duration of session was appropriate, respectively. Session participants significantly improved in perceived knowledge, skills and competencies (+ 8.6%; p = 0.025), and technical knowledge (+ 18.3%; p = 0.04) from baseline. Participating ARTc increased TB screening (+ 4.2%, p < 0.0001), TB diagnosis (+ 2.7%, p < 0.0001), ART initiation (+ 4.3%, p < 0.0001) and TB preventive treatment completion (+ 5.2%, p < 0.0001).

**Conclusion:**

Case-based e-learning is an acceptable and effective modus of capacity building and developing communities of practice to strengthen integrated care. E-learning could address demand for accessible and sustainable continuing professional education to manage complex diseases, and thereby enhance health equity. We recommend expansion of this initiative across the country for management of co-morbidities as well as other communicable and non-communicable diseases to augment the existing capacity building interventions by provide continued learning and routine mentorship through communities of practice.

## Background

With 2.35 million people living with HIV (PLHIV) [1] and 2.59 million annual tuberculosis incident cases [2], India has an estimated 53,000 HIV-TB co-infected people and 11,000 estimated deaths in 2020 [3]. The risk of developing tuberculosis may be 15–22 times greater in PLHIV [4], accounting for higher rates of mortality, causing one-in-three AIDS-related deaths [5]. Globally only 49% of people with HIV-TB were receiving treatment for both conditions [6] and co-management of HIV-TB coinfection continues to remain a challenge.

To strengthen collaboration between India’s National AIDS Control Programme (NACP) and National TB Elimination Programme (NTEP), the Government of India (GoI) launched single-window services for prevention and management of TB in PLHIV through Antiretroviral Therapy centres (ARTc) in December 2016 [7]. The overall aim of single window service delivery was to provide optimum care to HIV-TB co-infected persons. With the revised guidelines, the role of ARTc staff expanded to include TB management (which was hitherto done by NTEP). This necessitated to have a unified capacity building initiative for co-management of HIV-TB infection for ARTc staff which was previously deficient [8]. Implementation of integrated service delivery was further compounded by having two separate vertical national programmes for HIV and TB with separate capacity building and programme management systems. Lack of skilled health care workers, inadequacies of health systems, limited coordination for referral have been highlighted as major challenges for delivery of optimal TB care in other countries as well, particularly for HIV-TB [9, 10].

Therefore, case-based learning initiative for mentoring and capacity building using ECHO (Extension for Community Healthcare Outcomes) platform, was designed to build communities of practice to strengthen HIV-TB care at ARTc. This initiative is a virtual, case-based learning model designed to build capacity of ART Centres healthcare providers for HIV-TB case management through biweekly sessions using the ECHO platform. Case based e-learning is anchored on the principle of moving knowledge instead of patients by linking less-experienced providers with subject matter experts.

Globally, this is the first ever innovation which dealt with two different diseases and trained health care providers on integrated management of HIV-TB through case-based e-learning. The ECHO model has been successfully used in Presidents Emergency Plan for AIDS Relief (PEPFAR) supported countries to scale up HIV treatment [11, 12] and is also being used for mentoring the physicians engaged in managing TB and district functionaries of the NTEP in India.

In May 2018, NACP rolled out this case-based e-learning initiative to strengthen HIV-TB care at 115 ARTc that provided care to 391,264 PLHIV. Here, we describe the feasibility and acceptability of the case-based e-learning for HIV-TB care and its impact on professional satisfaction, self-efficacy (knowledge, skills and competency), technical knowledge retention (short-and long-term) and change in practice among HIV-TB care providers at ARTc in India.

## Methods

### Study type, population and setting

This quasi-experimental study included a cohort of ARTc staff invited to participate in case-based e-learning. NACP continues to provide ART to 1.4 million PLHIV and HIV-TB single-window services for co-infected persons through 540 ARTc that are primarily located in the public health institutions. National Institute of Tuberculosis and Respiratory Diseases (NITRD), moderated sessions as hub and 115 ARTc from four states (Delhi, Uttar Pradesh, Andhra Pradesh and Tamil Nadu) participated as spokes. Subject matter experts (referred to as experts) on HIV and TB were drawn both from NACP and NTEP, who provided expert opinion and facilitated case discussion.

### Sample size

**Fig. 1:**
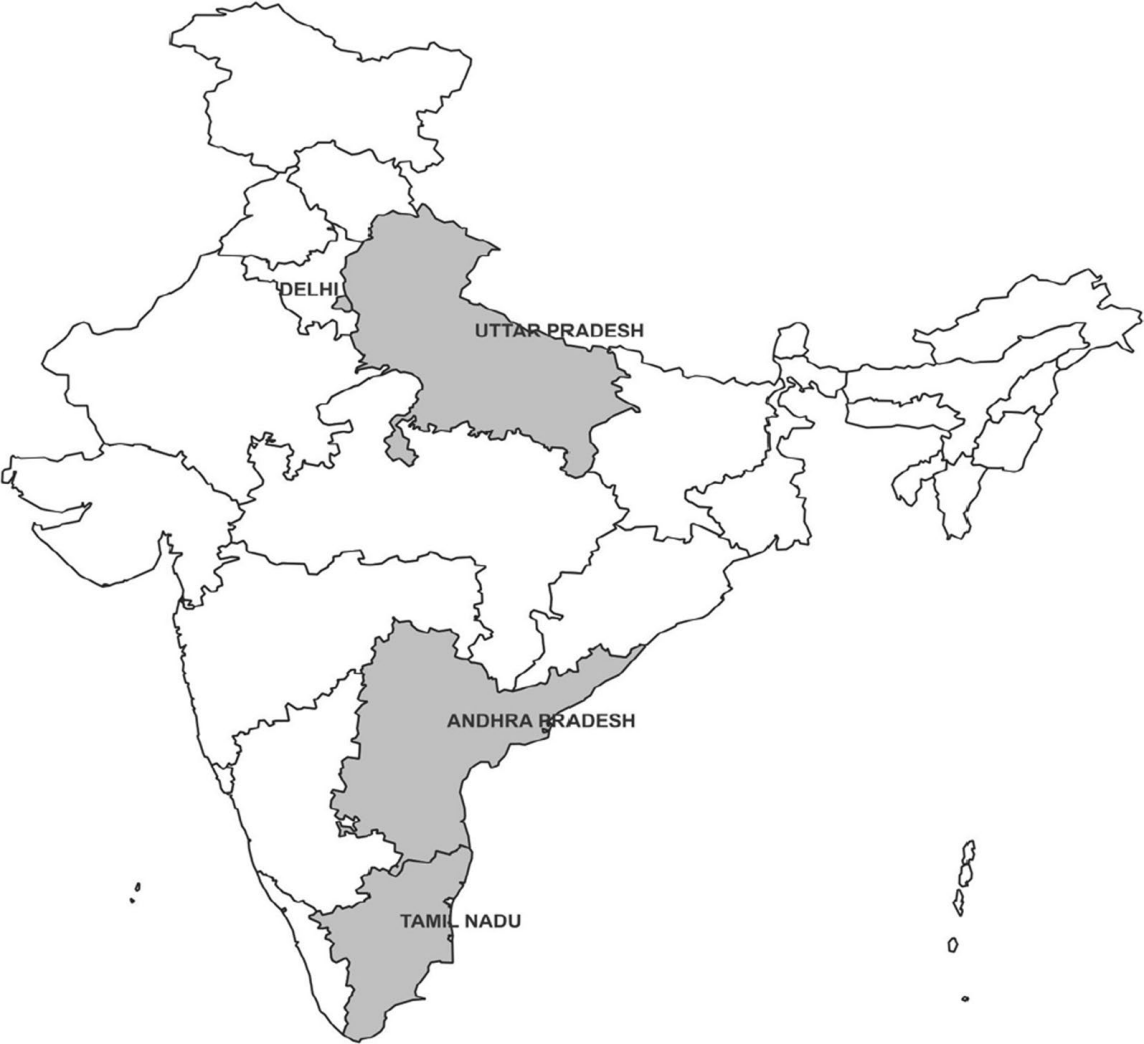
States where case-based e-learning was implemented. (Source: https://www.mapsofindia.com/)

We purposively selected two states (Andhra Pradesh, Tamil Nadu) with a high HIV prevalence (> national average of 0.22%) and two states (Delhi, Uttar Pradesh) with low HIV prevalence (< national average) to implement the intervention at all ARTc in the above states [11]. Geographically, the selected states represented two states from northern part of India and two states from southern part (Fig. 1). ART centres in selected states included high patient load (> 2000 PLHIV on ART) and mid patient load (500–1999 PLHIV on ART). In total, 115 of 540 (21%) ARTc functional across the country participated in the initiative. Participating cadre from each of the 115 ARTc included medical officer, nurse, counsellor, pharmacist and other cadres (data manager, care coordinator and lab technician) as per the staffing pattern at the ARTc in accordance with the national guidelines. Assuming 100% participation from each of 115 ARTc, the study had a power of 93.56%, 99.14% and 99.93% to detect improvement in knowledge score of 15%, 20% and 25% respectively with 95% confidence.

### E-learning intervention

This intervention was anchored on the conceptual framework of building communities of practice, defined as “groups of people who share a common concern/problems/passion, and deepen their knowledge and expertise by interacting on an ongoing basis” [14]. We developed curriculum based on the national TB prevention and management guidelines for PLHIV [7], in consultation with experts from both the programmes. We ensured availability of a computer, web camera, speaker with microphone, internet connectivity as per guidelines. We provided an orientation to the participating staff at the spokes on the functioning of case-based e-learning through a virtual platform, joining the meetings, participation in discussions, sharing files during the session, use of chat box, raise hand options for asking questions. We took feedback on preferred session day/timing to facilitate attendance.

The spokes shared difficult-to-manage cases in a standard format that captured clinical and laboratory details and the key queries about the case management. During the one-hour sessions, a predetermined spoke presented the case, followed by case discussion/didactic and proposed recommendations for case management by the invited subject experts.

### Data collection and management

Data was collected to understand– (1) feasibility and acceptability of the case-based e-learning; (2) impact on professional satisfaction, self-efficacy scoring (knowledge, skills and competency), technical knowledge retention (short-and long-term) and change in practices among providers. We referred to the Revised Standards for Quality Improvement Reporting Excellence (SQUIRE 2.0) guidelines from EQUATOR network which provide framework for reporting quality improvement in healthcare [15].

#### Feasibility and acceptability of e-learning

Feasibility and acceptability was assessed by using in-session information on date/time, participation, topic and expert. Each session concluded with a poll which captured feedback on four variables**–**quality, clarity, relevance of content, appropriateness of duration; and overall rating of the session which allowed open-ended comments from participating sites. Feedback regarding the appropriate topics, interaction of the participants, internet connectivity, quality of case presentation by spokes, relevance of case study, timeliness and effectiveness of e-learning using a structured questionnaire was collected from the experts and response were classified as Agree, Neutral and Disagree.

#### Impact of e-learning

Impact was analysed using three parameters**–**self-efficacy (an individual's belief in his or her capabilities to produce specific performance attainments), knowledge retention, and change in practices and patient outcomes. To assess *self-efficacy* (knowledge, skills and competencies), each spoke was subjected to an electronic survey at baseline and completion (at the end of 18 months). The scales used for assessment of self-efficacy were adapted from similar protocols used for evaluation of ECHO projects in other countries [16, 17]. *Short-term technical knowledge retention* was assessed through pre-and post-session assessment (immediately at the end of each session) whereas *long-term technical knowledge retention* was assessed through a structured electronic questionnaire pertaining to technical content in the national guidelines, at baseline and completion (at the end of 18 months). We reviewed routine programmatic data to assess practices among participating ARTc.

### Data analysis

*To assess feasibility of e-learning*, we calculated percentages for categorical variables using in-session data. We categorised sessions into five domains–diagnosis, treatment, prevention, pharmacological aspects and monitoring. Session participation, participant feedback was disaggregated into 5 domains. Responses from participants and experts on session feedback was collected on five-point scale and responses were re-categorised into three groups–Agreed, Neutral and Disagreed. ‘Agreed’ response rate for each session by feedback variable was calculated. A session was classified to have received as positive feedback if the agreed response rate was ≥ 80% for a specific variable. We calculated percentage of positive response for each survey question from experts. We compiled all participants’ feedback and included those pertaining to quality and relevance of the sessions for analysis.

*To assess impact of e-learning,* responses to ten self-efficacy questions through baseline and completion survey were collected on seven-point Likert scale for each question. Responses were re-categorised into three groups–Agreed, Neutral and Disagreed. Perceived mean scores of baseline and completion surveys were calculated and a paired t-test was used to determine the change in the perceived self-efficacy. For calculating the short-term technical knowledge retention, we used t-test to determine the mean difference in pre-and post-test scores administered during each session. For calculating the long-term knowledge retention, paired t-test was used to determine the mean difference in baseline and completion scores before and after 18 months of the intervention. p value < 0.05 was considered as significant. To estimate improvement in practices, we stratified sites by patient load and used chi- square test to measure change in process and patient outcomes–TB screening, TB referral, TB diagnosis, ART initiation and TB preventive treatment (TPT) completion rates over time.

## Results

Between May 2018 and September 2020, 59 e-learning sessions were conducted, spanning across five domains–diagnosis [n = 10(17%)], treatment [n = 27(46%)], prevention [n = 8(14%)], pharmacology [n = 8 (14%)] and monitoring [n = 6 (10%)].

### Feasibility of e-learning

**Fig. 2:**
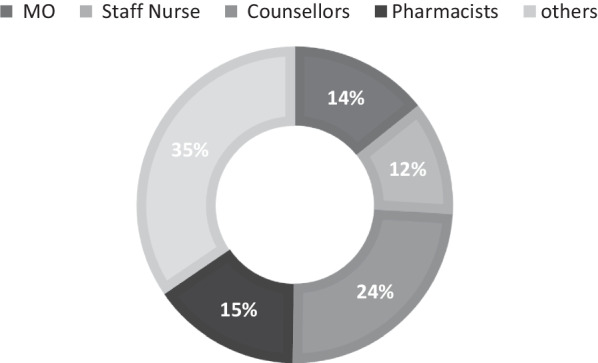
Mean participation of the spokes in e-learning sessions stratified by staff cadre (N = 152). *Cadres included in the “others” category were data manager, lab technician and care coordinators

**Fig. 3:**
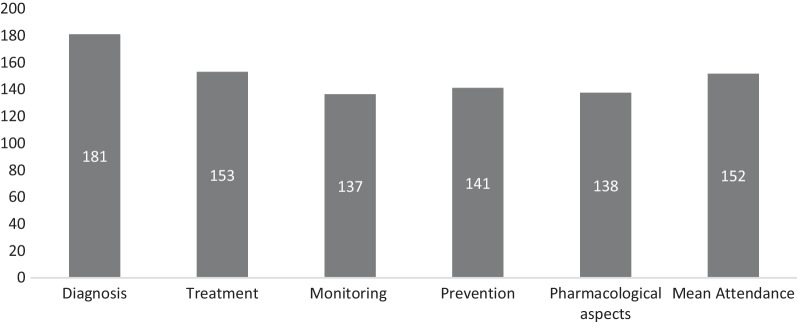
Mean participation of the ART centre staff in e-learning sessions stratified by the curriculum domain

The mean number of participating spokes per session was 55 and 100% spokes were retained until the completion of the study period. Each session had an average of 152 participants (range: 95–281) of which 16% were medical officers, 13% nurses, 26% treatment counsellors, 17% pharmacist and 38% were others [data manager, lab technician and care coordinators] (Fig. 2). Forty-eight percentage of the participants were females, and the median age of the participants was 39 years. All the staff except the care coordinator and laboratory technician had at least a graduate degree. Majority (82%) of the participants were working with NACP for more than 1 year. Participation was observed to be highest in sessions related to TB diagnosis (n = 181) and treatment (n = 153) (Fig. 3). Logistic issues leading to interruptions of session were reported for 2/59 (3%) sessions and were mainly internet related. For 81% of sessions, ≥ 80% of respondents found the session addressed specific queries they had; for 95% of sessions, ≥ 80% of the respondents agreed that topics were relevant to practice as well as had clarity and ease of understanding and; for 88% of sessions, ≥ 80% agreed that the duration of session was appropriate (Table 1).

**Table 1:** Distribution of sessions by positive feedback response rate in each session

	Positive feedback response rate	No. of sessions	%
Specific queries were addressed in the session	≥ 80%	48	81.36
	< 80%	11	18.64
Topic was relevant to practice	≥ 80%	56	94.92
	< 80%	3	5.08
Session had clarity and ease of understanding	≥ 80%	56	94.92
	< 80%	3	5.08
Session duration was appropriate	≥ 80%	52	88.14
	< 80%	7	11.86

Overall participants feedback received during in-session discussions included comments such as: “topic was work related”; “didactic presented in a way, which kept us awake even after lunch”; “information provided in fine sequence”; “sessions provide an opportunity to discuss the complex HIV-TB cases with experts, while sitting at the ARTc”; “we can choose the cases and then discuss them as per our priority”; “sessions provided insight on clinical as well as operational and data management aspects”; “sessions updated us on HIV-TB national guidelines”; “our queries were also solved on the treatment guidelines for paediatrics”; “great medium to learn best practices from other ARTc”; “we are thankful to our ART colleagues for sharing their cases with us”.

**Fig. 4:**
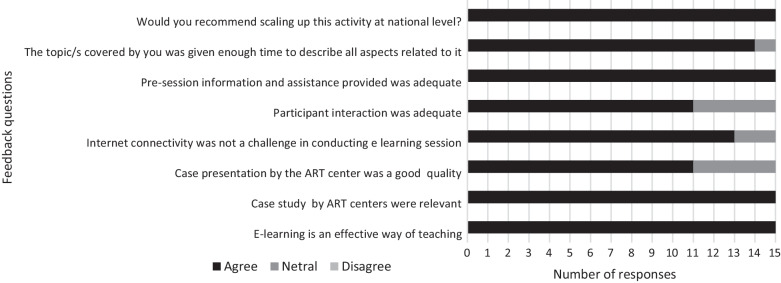
Expert response to survey on case-based e-learning initiative

Of the 15 experts who responded to the survey, 11(73%) had facilitated 2 or more e-learning sessions. All the experts (100%) agreed that e-learning was an effective modus of capacity building of staff spread across various geographies; the case studies presented by the ARTc were relevant; pre-session information and assistance was adequate; and opined that the e-learning initiative should be scaled up. Whereas 87% responded that internet connectivity was not a challenge; 73% thought participant interaction to be adequate and case presentation by the ARTc of good quality and; 93% agreed that format and time for the session were appropriate and adequate (Fig. 4).

Feedback from the session experts included comments such as: “a promising initiative to help field staff across different geographies”; “a good initiative to be scaled”; “it's a pathbreaking in terms of imparting pertinent clinical knowledge”; “helps in building communities of practice” and “overall a good initiative”; “need to expand the scope to non-communicable diseases”.

### Impact of e-learning

72/115 (63%) of spokes responded both for the baseline as well as the completion survey for assessment of self-efficacy (knowledge, skills and competencies) and long-term knowledge assessment.

#### Self-efficacy 

Perceived ease of access to experts and perceived ability to share clinical expertise with colleagues from other ARTc increased by 13% and 6% respectively. An increase of 8.6% (p = 0.025) was reported in self-efficacy (perceived knowledge, skills and competencies) on self-assessment, particularly with reference to ability to identify symptoms of TB in PLHIV (9.8%, p = 0.03), ability to diagnose and treat complicated cases of HIV-TB (11.4%, p = 0.05) and ability to assess and manage other co-morbidities in patients with HIV-TB (8.9%, p = 0.02) (Table 2).

**Table 2:** Improvement in self-efficacy before and after the e-learning initiative (Perceived mean score)

Question	Mean (Baseline)	SD (Baseline)	Mean (Completion)	SD (Completion)	p-value*	% Improvement
Identify symptoms of TB in PLHIV?	5.1	1	5.6	1.3	0.03	9.80%
Diagnose and treat TB in PLHIV (adults and adolescents)?	5.1	1	5.3	1.2	0.5	3.90%
Provide prophylaxis for TB (INH) in PLHIV (adults and adolescents)?	5.2	1	5.4	1.2	0.3	3.80%
Diagnose and treat TB in children living with HIV/AIDS?	4.6	1.3	4.7	1.4	0.5	2.20%
Provide prophylaxis for TB (INH) in children living with HIV/AIDS?	4.7	1.2	5	1.3	0.2	6.40%
Diagnose and treat complicated cases of HIV-TB?	4.4	1	4.9	1.5	0.05	11.40%
Assess and manage other co-morbidities in patients with HIV-TB?	4.5	1	4.9	1.3	0.02	8.90%
Educate and motivate HIV-TB co-infected patient for TB prevention and treatment retention?	5.2	1.1	5.5	1.1	0.15	5.80%
Recognize and manage side effects of ARV and anti TB medicines for all patients?	4.8	1.1	5.1	1.3	0.11	6.30%
Manage TB in patients with ART treatment failure?	4.7	1.1	4.9	1.5	0.24	4.30%

*A paired t-test was used to determine the significance (p ≤ 0.05)

#### Technical knowledge retention

Mean pre-and post-session scores to assess short-term technical knowledge retention indicated 16.2% (CI: 13.2–18.9, p < 0.001) improvement in technical knowledge score across the domains. Spokes who responded to both baseline and completion survey on long-term technical knowledge retention (n = 72/115), reflected an overall improvement in correct responses to technical questions by 18.3% (p = 0.04) from baseline.

#### Practices and patient outcomes

There was an increase in four-symptom screening by 4.2% (CI: 4.1–4.2, p < 0.0001); referrals for TB diagnosis by 2.0% (CI: 1.5–2.3, p < 0.0001); TB diagnosis by 2.7% (CI: 2.4–3.0, p < 0.0001); ART initiation by 4.3% (CI: 3.8–4.8, p < 0.0001), TPT completion by 5.2% (CI: 5.0–5.5, p < 0.0001). There was no significant difference in practices and patient outcomes between high and mid patient load ARTc.

## Discussion

To address gaps in HIV-TB management, innovative means of service delivery, complemented by continuous capacity building of staff are critical. The study evaluated the feasibility, acceptability, and effectiveness of case-based e-learning for HIV-TB care among providers at ARTc. The findings indicate substantial improvement in knowledge of participating spokes across all curriculum domains and a significant increase in self-efficacy, satisfaction, acquisition of knowledge and new skills.

All categories of staff were interested in sessions, corroborating that the case-based e-learning sessions as an acceptable means of team learning for ART centres’ staff and for building communities of practice. Participation of non-clinical healthcare providers in the e-learning initiative suggests increase in ownership and accountability by engaging them in development of session topics and presenting didactic lectures and cases. Participants reported most of the sessions to be clear with appropriate content, suitable timing and seamlessly broadcast without technical issues. The improvement in knowledge was translated into clinical management of TB as indicated by increase in symptom screening, diagnostic referral, TB treatment initiation and TPT completion rates.

Like other ECHO projects, our study demonstrated the ability of the HIV-TB e-learning initiative to improve healthcare worker knowledge and satisfaction and decrease professional isolation [16–18]. E-learning pedagogy provided participants an opportunity for active interaction and cross-learning among peers, thereby building communities of practice as observed in other case-based e-learning initiatives [19, 20]. The flexibility to revise the curriculum based on participant feedback and dynamic changes in the treatment and monitoring protocols is another advantage of the e-learning initiative for real-time content and with positive impact on patient outcomes. Findings reflect that it is feasible to provide continuous capacity building and mentoring of field staff without travel or suspension of clinical services in a cost-efficient manner as reflected in other similar interventions [21–24].

Experts engaged in this intervention found e-learning as an effective and interactive means for capacity building of staff distributed across remote geographies and suggested expansion to other geographies and disease programmes [25, 26].

The e-learning initiative facilitated both short-and long-term retention of participant knowledge, which was comparable to other similar interventions [27, 28], though most training methodologies have limitation to evaluate the long-term retention in Indian context.

### Limitations

ARTC were selected on a convenience and not on the basis of population, as this is considered a programmatic activity by NACO. Improvement in knowledge, practices and patient outcomes was measured by participating sites and not at individual level count, in accordance with the study objectives. The scales used for assessment of self-efficacy were adapted from similar protocols used for evaluation of ECHO projects in other countries, with no specific validation for this project. Implementation coincided with COVID surge, leading to competing priorities for health care providers which influenced their participation and therefore impact may have been more than what we were able to measure.

## Conclusion

The communities of practice developed through this intervention have decreased provider isolation and increased provider satisfaction, especially in light of current high staff turnover. This initiative garnered combined expertise from TB/HIV programmes to seek timely guidance by ARTc staff on complex queries related to prevention and management of HIV-TB. Findings recommend expansion of this initiative across the country to augment the existing capacity building interventions and engage other cadres who could potentially benefit from routine mentorship and continued professional development. Resonating well with lessons learnt during COVID-19 pandemic regarding use of technology, this initiative not only promoted health equity by timely provision of expert opinion but also kept the peripheral sites motivated by reducing professional isolation, even during pandemic times. Scale-up of this e-learning initiative by national programmes could help address the demand for accessible, cost-efficient and sustainable continuing professional education for management of other communicable and non-communicable diseases and offer an opportunity for preparedness to provide continued learning through communities of practice, even during unprecedented restrictive scenarios in future.

## Citation

Reshu Agarwal, Upasna Agarwal, Chinmoyee Das, Ramesh Allam Reddy et al.: Building communities of practice through case-based e-learning to prevent and manage TB among people living with HIV–India.

## CDC Authorship Disclaimer

The findings and conclusions in this report are those of the authors and do not necessarily represent the official position of CDC or other affiliated organisations.

## Data Availability

The datasets used and/or analysed during the current study are available from the corresponding author on reasonable request.

## Abbreviations

ARTc: Antiretroviral therapy centre
CDC: US Centres for Disease Control and Prevention
CI: Confidence interval
ECHO: Extension for Community Healthcare Outcomes
e-learning: Learning through digital platform
GoI: Government of India
NACO: National AIDS Control Organization
NACP: National AIDS Control Programme
NITRD: National Institute of Tuberculosis and Respiratory Diseases
NTEP: National Tuberculosis Elimination Programme
PEPFAR: President’s Emergency Plan for AIDS Relief
PLHIV: People living with HIV
TPT: TB preventive treatment
